# Investigation of the Potential Neuroprotective Mechanisms of *Acalypha indica* Against Alzheimer’s Disease by Integrated Bioinformatics Analysis

**DOI:** 10.3390/ijms27146196

**Published:** 2026-07-11

**Authors:** Ly Thi Huong Nguyen, Huong Thi Nguyen, Thai Uy Nguyen

**Affiliations:** 1 Institute of Theoretical and Applied Research, Duy Tan University, Hanoi 100000, Vietnam; nguyenthuongly5@duytan.edu.vn; 2 College of Medicine and Pharmacy, Duy Tan University, Da Nang 550000, Vietnam; 3 Cancer Research Program, Department of Medicine, McGill University Health Center, Montreal, QC H4A3J1, Canada; thi.nguyen22@mail.mcgill.ca; 4 College of Health Sciences, Vin University, Hanoi 100000, Vietnam; 5 Vinmec International Hospital, Hanoi 100000, Vietnam

**Keywords:** *Acalypha indica*, Alzheimer’s disease, bioinformatics, network pharmacology, molecular docking, gene set enrichment analysis, immune cell infiltration analysis

## Abstract

Alzheimer’s disease (AD) is one of the most common neurodegenerative disorders; however, available treatments majorly offer symptomatic relief without delaying disease progression and are associated with various adverse effects, highlighting the need for development of alternative therapies. *Acalypha indica* has previously showed neuroprotective effects in aging-related animal models, yet its mechanisms against AD were not fully understood. In this study, we employed an integrated bioinformatics approach combining network pharmacology, transcriptomic analysis, and molecular docking to investigate the anti-AD potential of this herb. A total of 282 overlapping targets between *A. indica* compounds and AD were identified. Network pharmacology analysis indicated chrysin, daidzein, galangin, kaempferol, and quercetin as the key bioactive components. Enrichment analyses suggested that targets of these compounds are mainly associated with phosphoinositide 3-kinase/protein kinase B (PI3K/Akt) and mitogen-activated protein kinase (MAPK) signaling pathways. Protein–protein interaction (PPI) analysis identified AKT1, epidermal growth factor receptor (EGFR), interleukin 6 (IL6), tumor necrosis factor (TNF), and p53 protein (TP53) as crucial hub targets. These targets were significantly upregulated in AD brain samples and were closely associated with pathways related to neurodegeneration, inflammation, as well as alterations in immune cell infiltration. Among the compounds, quercetin exhibited the strongest binding affinity to these target proteins. Overall, these findings provide a strong foundation for the multi-target therapeutic potential of *A. indica* in AD.

## 1. Introduction

Alzheimer’s disease (AD) is a progressive neurodegenerative disorder and the leading cause of dementia, characterized by memory loss and cognitive decline [1]. The hallmarks of AD include the accumulation of amyloid-beta (Aβ) plaques and neurofibrillary tangles (NFTs) composed of hyperphosphorylated tau proteins. These pathological changes disrupt synaptic function, impair neuronal plasticity, trigger microglial activation, and ultimately lead to neuroinflammation and neurodegeneration, particularly in the hippocampus and cerebral cortex [2]. Aging is considered as the most significant risk factor in the development of AD, with remarkably high incidences of AD-related neuropathological changes observed in the elderly population. The prevalence of these changes is approximately 22% in individuals aged ≥50 years, increasing to 33.4% in those aged ≥70 years [3,4]. Currently, cholinesterase inhibitors, such as donepezil, rivastigmine, and galantamine, are the most commonly used pharmacological treatments for AD. However, these medications primarily offer symptomatic improvement, with limited effectiveness in delaying disease progression [5]. Moreover, prolonged administration of these drugs is associated with various adverse events, such as nausea, diarrhea, anorexia, and insomnia [6], highlighting the need to develop alternative therapies for AD with better effectiveness and fewer side effects.

Herbal medicines have attracted increasing attention as alternative and complementary therapies for AD, with fewer adverse effects than conventional medicines. Clinical studies have shown that *Ginkgo biloba* extract improves cognitive function and memory in patients with early-stage AD, as demonstrated by significant improvements in cognitive assessment scores [7]. Moreover, *Panax ginseng* combined with conventional therapy demonstrated greater amelioration of cognitive impairment than standard treatment alone, suggesting its potential as an adjunctive treatment for AD [8]. Medicinal herbs consist of various bioactive phytochemicals that possess a wide range of biological activities by regulating multiple targets and signaling pathways, suggesting that their beneficial effects on AD may involve complex underlying mechanisms of action [9]. In this context, integrated bioinformatics approaches that combine multiple analyses, such as compound–target networks, protein–protein interaction networks, pathway enrichment analysis, and molecular docking, can provide a systematic exploration of the molecular targets and signaling pathways underlying the anti-AD potential of herbal medicines.

*Acalypha indica* L. is a common traditional herbal medicine in Asia, particularly in India and Southeast Asia. It has been widely used to treat various symptoms and diseases, such as respiratory diseases (bronchitis, pneumonia, and asthma) and skin disorders (eczema and psoriasis) [10,11]. *A. indica* possesses a broad spectrum of biological and pharmacological properties, including anti-inflammatory, antioxidant, antibiotic, antitumor, and wound-healing effects. An ethanolic extract of *A. indica* root exhibited neuroprotective effects against aging by increasing brain-derived neurotrophic factor (BDNF) levels and reducing malondialdehyde (MDA) levels in the brains of old rats [12,13]. In addition, a water extract of *A. indica* roots demonstrated neuroprotective and neurotherapy effects in frogs [14], while a methanolic extract of *A. indica* leaves exhibited anticonvulsant activity in FeCl_3_-induced epilepsy in Sprague Dawley rats [15]. Similarly, a methanolic whole plant extract also exerted antiepileptic and anxiolytic effects Wistar albino rats, possibly through modulation of the gamma-aminobutyric acid (GABA)ergic system by acting as a potential GABA agonist [16]. Moreover, *A. indica* exerts beneficial effects on motor function impairment and anxiety-like behaviors in 1-methyl-4-phenyl-1,2,3,6-tetrahydropyridine (MPTP)-induced mouse models of Parkinson’s disease [17]. However, the underlying molecular mechanisms through which *A. indica* exerts its potential therapeutic effects against AD have not yet been systematically investigated.

In the present study, we employed an integrated bioinformatics approach combining network pharmacology, molecular docking, and pharmacokinetic prediction to investigate the molecular mechanisms underlying the anti-AD potential of *A. indica*. The phytochemical compositions of *A. indica*, along with its potential anti-AD targets and associated signaling pathways, as well as the interactions between active compounds and target proteins were systematically analyzed. The findings of this study provide a valuable foundation for future investigations into the therapeutic potential of *A. indica* as an alternative or adjunctive therapy for AD.

## 2. Results

### 2.1. Screening for Target Genes of A. indica Compounds Against AD

**Figure 1:**
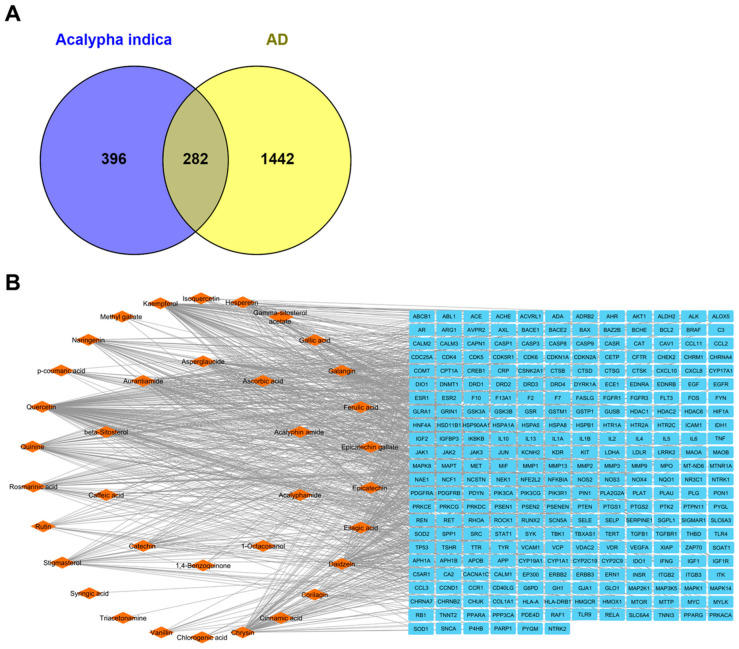
Network of common targets between *A. indica* compounds and AD. (**A**) Venn diagram showing the intersection between *A. indica* and AD-related targets. (**B**) Compound-target network of overlapping targets between *A. indica* and AD.

Using literature search and public databases (Dr. Using the Duke’s Phytochemical and Ethnobotanical database and the Indian Medicinal Plants, Phytochemistry, and Therapeutics (IMPPAT) database), 40 compounds of *A. indica* were retrieved for further investigation of their therapeutic potential in AD (Table S1). The Traditional Chinese Medicine Systems Pharmacology Database and Analysis Platform (TCMSP) and SwissTargetPrediction databases were used to identify 678 putative target genes associated with these compounds (probability > 0.1) (Table S2). The GeneCards, MalaCards, and OMIM databases revealed 1724 AD-related genes with relevance scores ≥ 20. Venny 2.1 was used to intersect these gene sets, and 282 common targets between *A. indica* compounds and AD-associated genes were identified (Figure 1A). A Venny 2.1 compound–target network was constructed using Cytoscape, consisting of 318 nodes (36 compounds and 282 targets) connected by 983 edges (Figure 1B). Detailed information on the chemical structures, physicochemical properties, pharmacokinetic profiles, and drug-likeness of these 36 compounds is provided in Table S3. Topological analysis of the network highlighted several key compounds with relatively high degree values, including quercetin, kaempferol, daidzein, chrysin, and galangin (Table 1), suggesting that these compounds may be the major bioactive constituents of *A. indica*, contributing to its potential therapeutic effects against AD.

### 2.2. KEGG and GO Enrichment Analysis of Potential Anti-AD Targets

**Figure 2:**
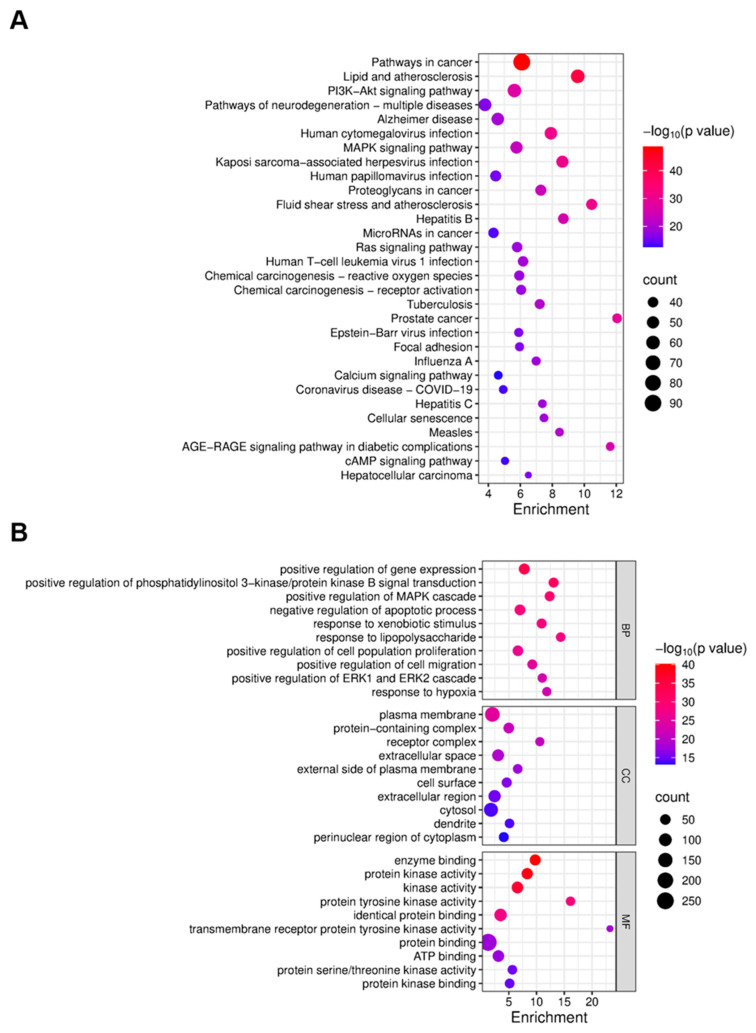
Enrichment analysis of potential targets. (**A**) KEGG pathway analysis. (**B**) GO enrichment analysis. BP: biological process; CC: cellular component; MF: molecular function.

KEGG pathway enrichment analysis suggested that the targets of *A. indica* are associated with 199 signaling pathways (Table S4). Several pathways are implicated in AD pathogenesis, particularly the PI3K–Akt signaling pathway, pathways in neurodegeneration—multiple diseases, Alzheimer disease, and the MAPK signaling pathway (Figure 2A). The details of these enriched pathways, along with the corresponding predicted targets of *A. indica*, are illustrated in Figures S1–S4.

GO enrichment analysis identified 1299 biological processes (BPs), 160 cellular components (CCs), and 262 molecular functions (MFs) associated with the therapeutic targets of *A. indica* in AD (Tables S5–S7). The top 30 enriched GO terms are shown in Figure 2B. The most enriched BP terms were positive regulation of gene expression, positive regulation of phosphatidylinositol 3-kinase/protein kinase B signaling, and positive regulation of the MAPK cascade. The MF categories were primarily associated with enzyme binding, protein kinase activity, and kinase activity. In the CC category, the enriched terms were mainly related to the plasma membrane, protein-containing complexes, and receptor complexes.

### 2.3. Protein–Protein Interaction (PPI) Network

**Figure 3:**
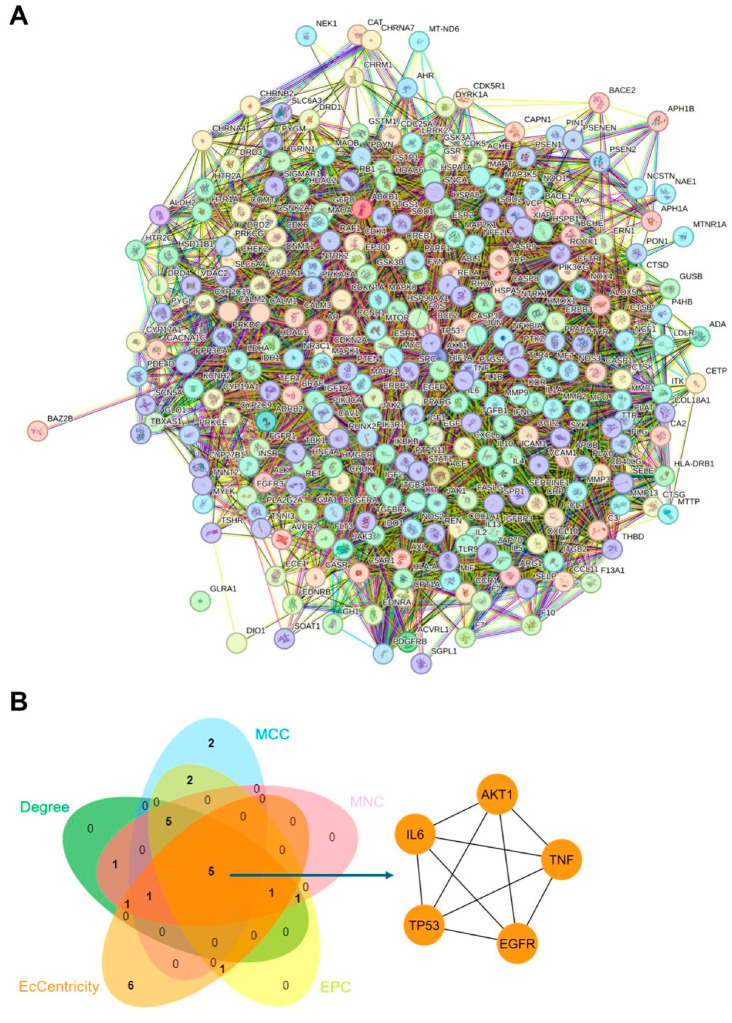
Protein–protein interaction (PPI) network analysis of potential therapeutic targets. (**A**) PPI network of the predicted anti-AD targets of *A. indica*. (**B**) Venn diagram illustrating the identification of five hub genes obtained from the intersection of the five CytoHubba algorithms.

Interactions between proteins play a crucial role in maintaining cellular homeostasis, and investigating these interactions can provide valuable insights into potential therapeutic targets for disease management and treatment. The STRING tool ver. 12.0 was used to map the 282 targets into a PPI network (Figure 3A). The network included 282 nodes and 8240 edges, with a PPI enrichment *p*-value < 10^−16^ and an average node degree of 58.4. Five hub proteins were identified by intersecting the top 15 proteins from the five different CytoHubba algorithms (MCC (maximal clique centrality), MNC (maximum neighborhood component), EPC (edge percolated component), Degree, and EcCentricity) (Figure 3B). The five key targets were AKT1, EGFR, IL6, TNF, and TP53, which were used for further analysis.

### 2.4. Expression and Diagnostic Performance of the Five Hub Genes

**Figure 4:**
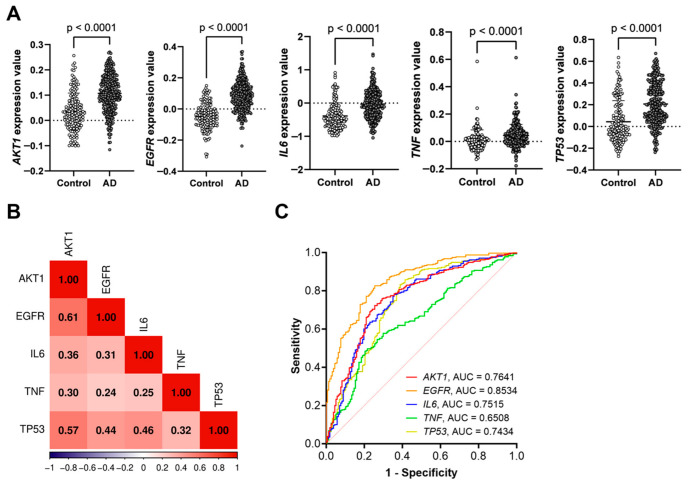
Expression and diagnostic performance of the five hub genes. (**A**) Expression of the five hub genes in the GSE33000 dataset. (**B**) Correlation analysis of the five hub genes. (**C**) Receiver operating characteristic (ROC) curve analysis of the diagnostic performance of the five hub genes.

The expression profiles of the five hub genes, *AKT1*, *EGFR*, *IL6*, *TNF*, and *TP53*, were analyzed in brain tissues from patients with AD and healthy individuals using the GSE33000 dataset (Figure 4A). The results showed that all five genes were significantly upregulated in AD samples compared to healthy controls. Pearson’s correlation analysis indicated positive correlations among all hub genes (*p* < 0.0001) (Figure 4B). *AKT1* exhibited the strongest positive association with *EGFR* (r = 0.61) and *TP53* (r = 0.57), suggesting the potential coordinated regulatory roles of these genes in AD progression. Moreover, receiver operating characteristic (ROC) curve analysis revealed that all five genes had promising diagnostic value for distinguishing patients with AD from healthy controls (Figure 4C). The AUC values were 0.7641 for *AKT1* (*p* < 0.0001), 0.8534 for *EGFR* (*p* < 0.0001), 0.7515 for *IL6* (*p* < 0.0001), 0.6508 for *TNF* (*p* < 0.0001), and 0.7434 for *TP53* (*p* < 0.0001). These findings indicate that the five hub genes could serve as potential diagnostic biomarkers and may contribute significantly to the molecular mechanisms underlying AD. Therefore, targeting these biomarkers with compounds derived from *A. indica* may help alleviate AD symptoms.

### 2.5. Single-Gene Gene Set Enrichment Analysis (GSEA) of Hub Genes

**Figure 5:**
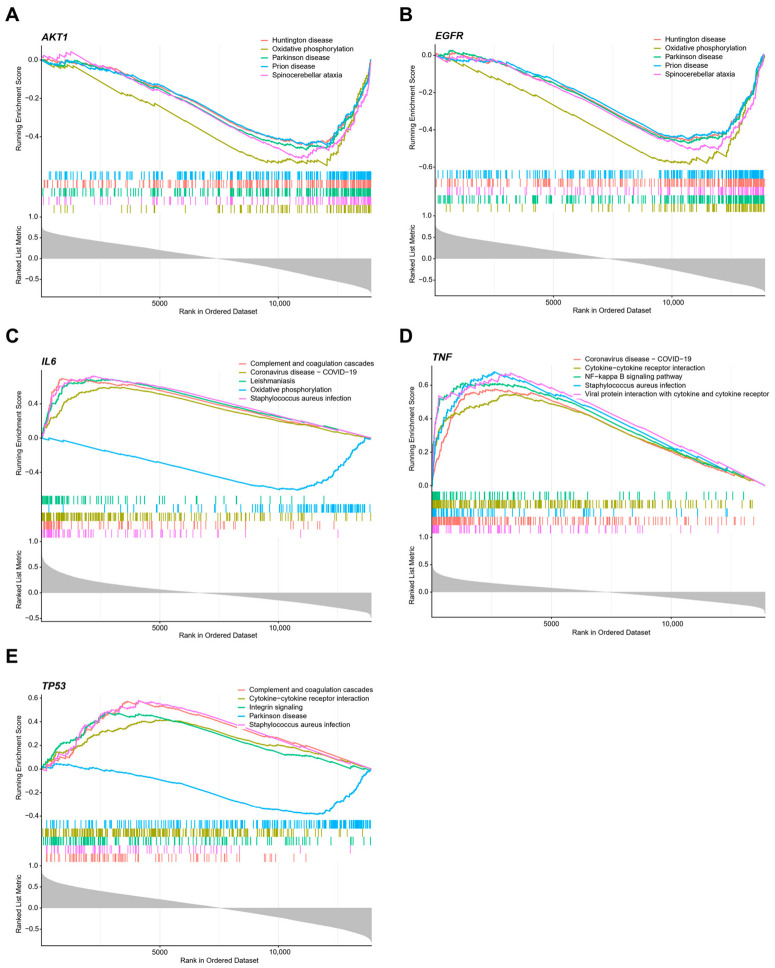
Single-gene GSEA-KEGG pathway analysis of five hub genes. (**A**) AKT1. (**B**) EGFR. (**C**) IL6. (**D**) TNF. (**E**) TP53.

Single-gene GSEA revealed that the five hub genes were closely associated with multiple signaling pathways related to AD in the GSE33000 dataset, as shown in Figure 5. *AKT1* and *EGFR* were predominantly enriched in neurodegeneration-related pathways, including Huntington’s disease, Parkinson’s disease, prion disease, spinocerebellar ataxia, and oxidative phosphorylation. *IL6*, *TNF*, and *TP53* were mainly associated with pathways related to inflammatory and immune responses, such as cytokine–cytokine receptor interaction, NF-κB signaling, complement and coagulation cascades, and integrin signaling. These results suggest that the five hub genes may contribute to AD development and progression by modulating pathways associated with neurodegeneration and neuroinflammation.

### 2.6. Immune Landscape and Hub Gene Correlation Analysis

**Figure 6:**
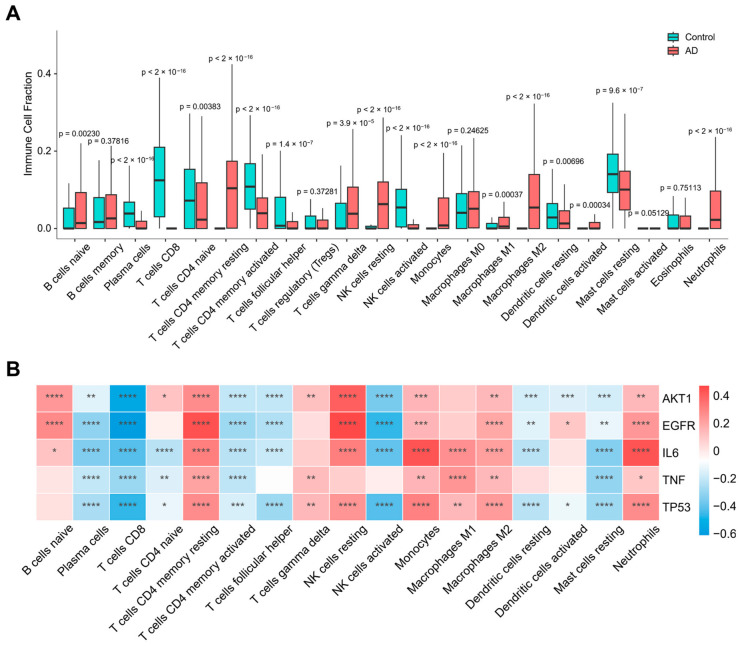
Immune cell infiltration analysis. (**A**) Differential distribution of 22 immune cell types between the AD and control groups. (**B**) Correlation analysis between the five hub genes and the significantly altered immune cell populations. * *p* < 0.05, ** *p* < 0.01, *** *p* < 0.001, **** *p* < 0.0001.

Immune infiltration analysis indicated significant alterations in the proportions of various immune cell types in the brain tissues of patients with AD compared to healthy controls. AD brain samples showed elevated infiltration of naïve B cells, resting CD4 memory T cells, gamma delta T cells, resting NK cells, monocytes, M1/M2 macrophages, activated dendritic cells, and neutrophils (Figure 6A). In contrast, plasma cells, CD8 T cells, activated CD4 memory T cells, follicular helper T cells, activated NK cells, resting dendritic cells, and resting mast cells were markedly decreased in the AD group (Figure 6A). These results imply that immune dysregulation is closely associated with AD and may contribute to its pathogenesis.

All five hub genes, *AKT1*, *EGFR*, *IL6*, *TNF*, and *TP53*, are closely associated with immune regulation and are involved in multiple signaling pathways governing immune responses and neuroinflammation in AD [18]. To further validate their immunological significance, Pearson’s correlation analysis was performed between the expression levels of these hub genes and the proportions of 17 immune cell types that were significantly altered between the AD and control groups in the GSE33000 dataset (Figure 6B). Our results showed that all five genes had positive correlations with monocytes, macrophages M2, neutrophils, and resting CD4 memory T cells. In contrast, these hub genes were negatively correlated with plasma cells, CD8 T cells, activated CD4 memory T cells, and resting mast cells. *IL6* demonstrated the strongest positive association with monocytes and neutrophils, whereas *TNF* and *TP53* exhibited comparatively weaker correlations. *AKT1* and *EGFR* exhibited similar correlation patterns, characterized by strong positive correlations with resting CD4 memory T cells and resting NK cells and remarkable negative correlations with CD8 T cells and activated NK cells. These findings suggest that the five hub genes may contribute to AD development and progression by regulating immune cell infiltration and inflammation.

### 2.7. Molecular Docking Analysis of Potential Compounds and Hub Targets

**Figure 7:**
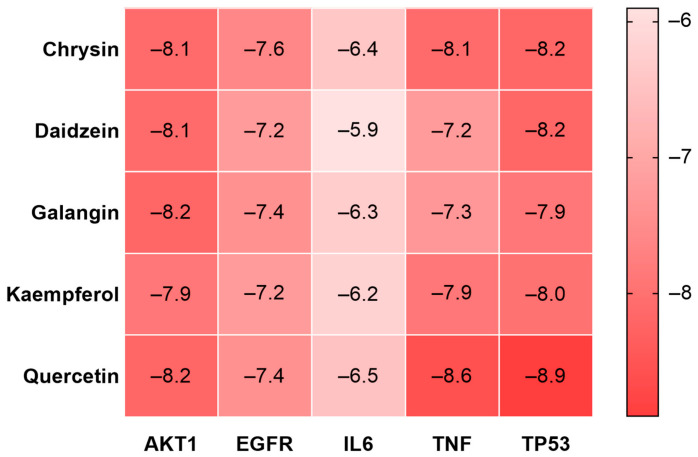
Docking scores between potential compounds and five hub proteins. A darker color indicates a lower binding energy for the interactions.

**Figure 8:**
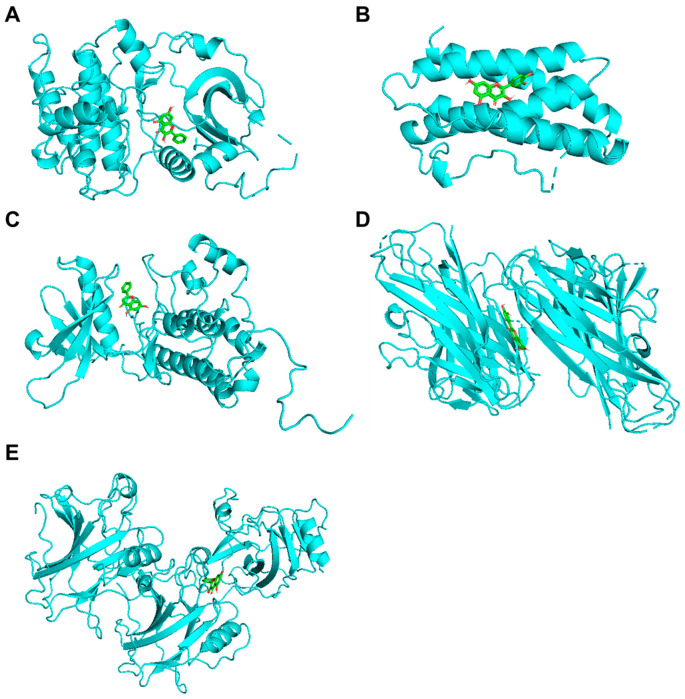
3D structures of the binding between potential compounds and hub proteins. (**A**) Galangin–AKT1 complex. (**B**) Chrysin–EGFR complex. (**C**) Quercetin–IL6 complex. (**D**) Quercetin–TNF. (**E**) Quercetin–TP53 complex. The cyan color represents proteins, while the green and red colors represent carbon and oxygen atoms in the compounds, respectively.

**Figure 9:**
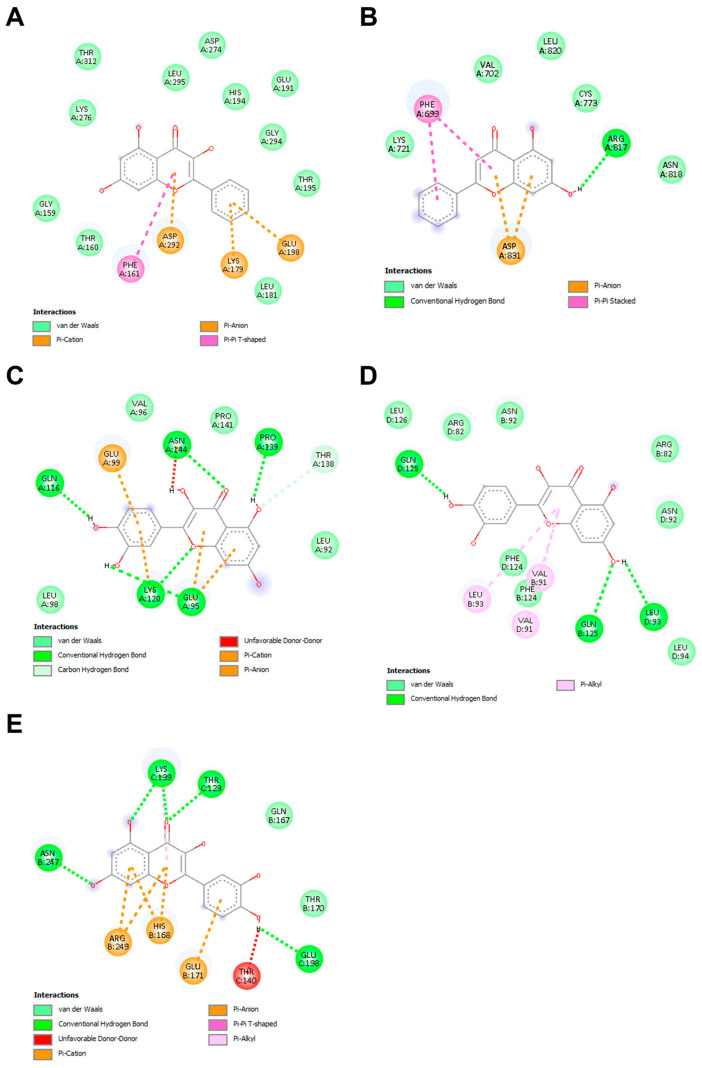
2D structures of the binding between potential compounds and hub proteins. (**A**) Galangin–AKT1 complex. (**B**) Chrysin–EGFR complex. (**C**) Quercetin–IL6 complex. (**D**) Quercetin–TNF. (**E**) Quercetin–TP53 complex.

Molecular docking analysis revealed potential favorable interactions between the five selected compounds (chrysin, daidzein, galangin, kaempferol, and quercetin) and the five hub proteins (AKT1, EGFR, IL6, TNF, and TP53) (Figure 7). Quercetin showed the strongest overall binding affinities with the hub proteins, particularly with TP53 (−8.9 kcal/mol) and TNF (−8.6 kcal/mol). Galangin and quercetin demonstrated the strongest interaction with AKT1 (−8.2 kcal/mol), whereas chrysin exhibited the highest affinity for EGFR (−7.6 kcal/mol). All compounds had moderate docking scores with IL6, ranging from −6.5 to −5.9 kcal/mol. The detailed 3D and 2D binding modes of the most favorable compound–protein complexes are shown in Figure 8 and Figure 9, respectively. Table 2 summarizes the interaction types, interacting residues, and corresponding bond distances identified in the representative protein–compound complexes. The interactions mainly involved van der Waals forces, conventional hydrogen bonds, and non-covalent interactions (π–anion and π–cation contacts) at the active sites of the complexes. These findings suggest that the five potential bioactive compounds of *A. indica* may effectively interact with AD-related hub proteins, with quercetin showing the greatest potential as a therapeutic candidate for AD.

### 2.8. Molecular Dynamics Simulation Analysis of Protein-Ligand Complexes

**Figure 10:**
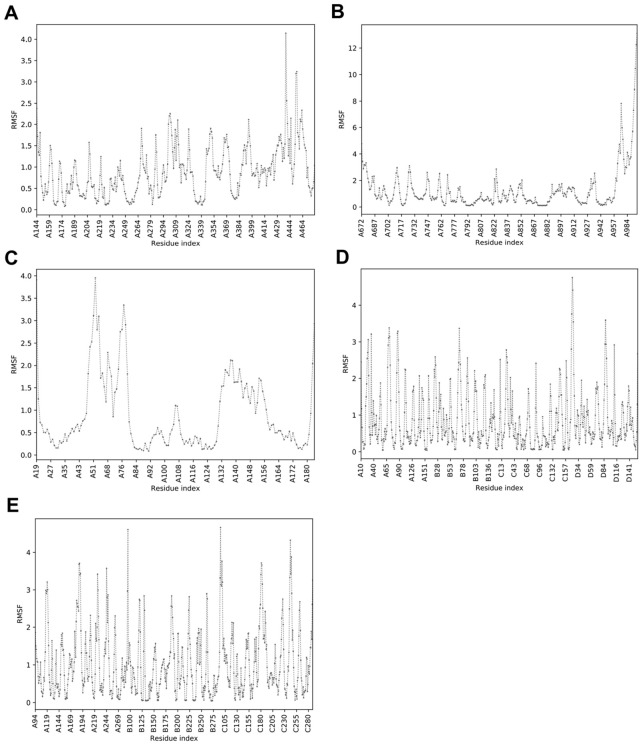
RMSF profiles of protein–ligand complexes obtained using CABS-flex 2.0. (**A**) AKT1–Galangin. (**B**) EGFR–Chrysin. (**C**) IL-6–Quercetin. (**D**) TNF–Quercetin. (**E**) TP53–Quercetin.

Molecular dynamics simulation was performed for the protein–ligand complexes exhibiting the strongest binding affinities, including AKT1–Galangin, EGFR–Chrysin, IL-6–Quercetin, TNF–Quercetin, and TP53–Quercetin. The minimum and maximum RMSF values with the associated residues are presented in Table 3, and the RMSF profiles are illustrated in Figure 10. Overall, most of the analyzed complexes exhibited RMSF values below 10 Å during the 10 ns simulation, indicating relatively stable protein–ligand interactions. However, the EGFR–Chrysin complex showed a comparatively higher maximum fluctuation value (13.102 Å), suggesting increased flexibility in specific regions of the protein during the simulation.

### 2.9. Pharmacokinetics Prediction of the Potential Compounds of A. indica

The pharmacokinetic profiles, including absorption, distribution, metabolism, excretion, and toxicity (ADMET), of the five selected compounds were predicted using the pkCSM tool to examine their potential as anti-AD agents (Table 4). In terms of absorption, chrysin, daidzein, and galangin exhibited high Caco-2 permeability values (log P_app_ > 0.9) and intestinal absorption rates (>93%), indicating strong intestinal permeability and absorption. In comparison, kaempferol and quercetin showed relatively lower Caco-2 permeability and intestinal absorption values, but were still acceptable for oral administration.

Prediction of distribution properties revealed remarkable differences in tissue distribution and brain penetration among the five compounds. Kaempferol and quercetin exhibited higher predicted volumes of distribution (VDss) (log(L/kg) > 1) and unbound fractions, suggesting a broader systemic distribution than that of the other compounds. Chrysin and daidzein exhibited relatively higher blood–brain barrier (BBB) and central nervous system (CNS) permeability values than the other compounds, indicating a greater potential to reach the brain and exert effects on AD. In contrast, quercetin exhibited the lowest BBB and CNS permeability values, suggesting a relatively limited ability to penetrate the brain tissue.

Cytochrome P450 (CYP) is a superfamily of enzymes that play crucial roles in the metabolism and detoxification of xenobiotics. Notably, members of the CYP1, CYP2, and CYP3 families, especially CYP1A2, CYP2C9, CYP2C19, CYP2D6, and CYP3A4, are responsible for the metabolism of over half of all clinically used drugs [19]. In addition, CYP2D6 and CYP3A4 play an important role in the metabolism of common drugs used for AD, including donepezil and galantamine [20]. Our analysis indicated that all five compounds were predicted to inhibit CYP1A2, whereas chrysin, daidzein, and galangin were also predicted to inhibit CYP2C19 and CYP2C9. However, none of the compounds showed inhibitory effects on CYP2D6 and CYP3A4, suggesting a relatively low potential for major drug–drug interactions associated with these metabolic enzymes. Excretion prediction indicated moderate total clearance values for all investigated compounds, with kaempferol exhibiting the highest predicted clearance rate (log(mL/min/kg) = 0.477). Organic cation transporter 2 (OCT2), which is majorly expressed in renal proximal tubule cells, plays an important role in the renal excretion of a wide range of prescription drugs [21]. None of the compounds were predicted to be substrates of renal OCT2, indicating that their excretion is unlikely to involve OCT2-mediated renal transport.

Importantly, toxicity prediction analysis indicated favorable safety profiles for all five compounds tested. None of the compounds exhibited predicted mutagenic or hepatotoxic effects, indicating that these compounds may be safe for administration. Furthermore, kaempferol and quercetin demonstrated relatively higher maximum tolerated doses, suggesting potentially better systemic tolerability compared to other compounds. Taken together, ADMET prediction suggests that the five potential compounds of *A. indica* exhibit promising pharmacokinetic profiles relevant to AD treatment. 

## 3. Discussion

AD is considered as a complex and multifactorial neurodegenerative disease caused by the interplay of various factors, including genetics, aging, and environmental factors. Moreover, multiple pathological mechanisms contribute to disease progression, including Aβ plaques, tau hyperphosphorylation, neuroinflammation, and vascular dysfunction [22]. This complexity in the pathogenesis of AD suggests that effective treatment strategies may require multi-target therapeutic approaches rather than mono-target therapies. Several treatments aimed exclusively at a single pathological contributor, such as Aβ or tau proteins, have demonstrated limited efficacy in improving AD symptoms or slowing disease progression, while some have failed to provide significant clinical benefits [23]. Therefore, herbal medicines containing multiple bioactive phytochemicals that can simultaneously regulate diverse molecular targets may offer a promising therapeutic approach for AD. This study indicates that *A. indica* contains numerous bioactive compounds with potential anti-AD effects by targeting multiple genes and signaling pathways. These findings are consistent with previous studies of medicinal plants, which have indicated that herbal medicines typically act through synergistic modulation of complex networks rather than single targets for AD treatment [24,25].

In this study, we identified 36 bioactive compounds from *A. indica* that collectively targeted 282 genes related to AD. These compounds are predominantly phenolic constituents, particularly flavonoids, such as quercetin, kaempferol, and chrysin, as well as phenolic acids, including caffeic acid, ellagic acid, and rosmarinic acid. Growing evidence has highlighted the therapeutic potential of flavonoids and phenolic acids in AD due to their anti-neuroinflammatory, antioxidant, and neuroprotective properties against Aβ- and tau-associated pathologies [26,27]. These findings support the potential anti-AD effects of herbal medicines rich in these bioactive compounds, including *A. indica*. Network pharmacology analysis revealed that chrysin, daidzein, galangin, kaempferol, and quercetin might be the most important components of *A. indica* in AD treatment, as indicated by their highest degrees in the compound–target network, demonstrating their broad therapeutic potential by targeting multiple AD-related genes. These five phenolic compounds are commonly found in many different medicinal plants, such as *Alpinia* species, *Glycine max*, and *Pueraria lobata*, and their broad spectrum of biological activities, including antioxidant, anti-inflammatory, and neuroprotective effects, have been widely reported in previous studies [28,29]. Notably, several preclinical studies have demonstrated the beneficial effects of these compounds on cognitive decline and memory impairment in both genetic and chemically induced animal models of AD by regulating multiple signaling pathways [30,31,32,33,34]. These findings align well with our in silico network analysis, supporting the reliability of the predicted compound–target associations in this study.

The five bioactive components of *A. indica* exhibited favorable pharmacokinetic profiles, as predicted by pkCSM. Among these, chrysin and daidzein showed higher BBB and CNS permeability, whereas kaempferol and quercetin had limited brain penetration abilities due to their higher molecular weights. However, several advanced brain-delivery strategies for kaempferol and quercetin, including solid lipid nanoparticles, magnesomes, and plasma-derived exosomes, have been developed to enhance their bioavailability in the brain and improve their therapeutic efficacy against AD [35,36,37]. In addition, polyphenols possess characteristic molecular structures containing aromatic rings and hydroxyl groups, which favor supramolecular co-assembly through hydrogen bonding and π–π stacking interactions [38]. Given the relatively high BBB permeability predicted for chrysin and daidzein, it is conceivable that these compounds may co-assemble with other polyphenols, such as kaempferol and quercetin, potentially facilitating their transport across the BBB and enhancing their brain delivery. However, no direct evidence has yet demonstrated that *A. indica*-derived assemblies can enhance brain penetration of quercetin and kaempferol. Accumulating evidence suggested that polyphenols can exert neuroprotective effects indirectly through regulation of peripheral metabolism and the gut–brain axis without requiring substantial central nervous system exposure [39]. Notably, quercetin nanoparticles demonstrated anti-AD effects in obesity- and aging-induced AD mice by increasing the abundance of anti-inflammatory gut microbiota, including *Akkermansia* and *Lactobacillus*, and attenuating neuroinflammation [40]. These findings highlight the potential of *A. indica*, which contains these bioactive polyphenols, as a promising candidate for AD treatment.

Enrichment analysis of the potential targets of *A. indica* provided insights into its possible mechanisms of action in AD. GO enrichment analysis in the cellular component category indicated that the targets of *A. indica* were predominantly associated with the plasma membrane, receptor complexes, and protein-containing complexes. These findings suggest that *A. indica* may exert its effects by involving ligand–receptor binding on the cell membrane and regulating intracellular protein–protein interactions involved in the activation of signal transduction pathways. In terms of biological processes and molecular functions, the bioactive compounds of *A. indica* mainly affect protein kinase activity, especially through the regulation of the PI3K/Akt and MAPK signal transduction. As discussed earlier, *A. indica* is rich in phenolic compounds that contain aromatic rings and hydroxyl groups, which are key structural features associated with kinase inhibitory activity through interference with the ATP-binding pocket within the catalytic domain of protein kinases [41]. This mechanism is consistent with previously published studies reporting that flavonoids and other polyphenols act as broad-spectrum inhibitors of kinase signaling cascades involved in inflammation and neurological disorders [42]. This may partly explain the regulatory effects of *A. indica* on protein kinase activity, particularly in the PI3K/Akt and MAPK signaling pathways.

Consistent with the GO enrichment results, KEGG pathway analysis also suggested that the targets of *A. indica* are involved in the regulation of the PI3K/Akt and MAPK signaling pathways. Under physiological conditions, the PI3K/Akt pathway plays a crucial role in the regulation of cell survival, proliferation, differentiation, apoptosis, and autophagy to maintain cellular homeostasis. However, in AD, this pathway is dysregulated and exerts dual and context-dependent roles in disease pathogenesis, which vary according to cell type and disease stage [43]. In the early stages of AD, autophagy plays a crucial role in clearing Aβ plaques. Inhibition of the PI3K/Akt signaling pathway, followed by suppression of mTOR activity, enhances autophagic flux and promotes Aβ degradation [44]. In contrast, during the later stages of AD, excessive accumulation of Aβ may aberrantly activate mTOR signaling, thereby impairing the normal autophagy capacity [45]. The role of the PI3K/Akt pathway in AD appears to be highly dependent on the cellular context. In neuronal cells, Aβ suppresses PI3K/Akt activation, leading to reduced cell survival and increased neuronal death. Inhibition of PI3K/Akt signaling further promotes the activation of glycogen synthase kinase-3β (GSK-3β), which induces tau hyperphosphorylation and NFT formation, ultimately contributing to tau-mediated neurotoxicity [46]. In contrast, activation of PI3K/Akt signaling has been observed in microglial cells in AD, where it is associated with neuroinflammation and oxidative stress, which contribute to neurodegeneration [47]. Several herbal medicines and natural compounds have been reported to exert therapeutic effects against AD by modulating the PI3K/Akt signaling pathway, either by inhibiting or activating its activity, depending on the pathological context. For instance, arctigenin derived from *Arctium lappa* alleviated memory impairment by enhancing autophagy-mediated Aβ clearance through suppression of the AKT/mTOR pathway, whereas oil extracted from *Acorus tatarinowii* improved learning and memory function via activation of the PI3K/Akt signaling pathway [48,49]. Collectively, these studies demonstrate that modulation of the PI3K/Akt signaling pathway represents a promising therapeutic strategy for AD, which is consistent with the pathway enrichment results obtained in our study. These findings indicate that *A. indica* may have therapeutic potential against AD through the regulation of the PI3K/Akt signaling pathway. In fact, naringenin, a flavonoid in *A. indica*, alleviated Aβ-induced neuronal damage by activating PI3K/Akt pathway in PC12 cells [50]. Similarly, caffeic acid, another component of *A. indica*, also showed neuroprotective effects via PI3K/Akt activation in Aβ-induced AD mice [51]. Nevertheless, the precise regulatory effect of *A. indica* on this pathway, whether through activation or inhibition, remains unclear and requires further investigation.

In addition to the PI3K/Akt signaling pathway, the MAPK pathway was significantly enriched among the potential targets of *A. indica*. MAPK signaling plays a central role in regulating various cellular processes, including cell proliferation, differentiation, apoptosis, and inflammatory responses, under both physiological and pathological conditions. In AD, the accumulation of Aβ can activate the p38 MAPK signaling pathway in microglial cells, leading to increased production of pro-inflammatory cytokines such as IL-1β, IL-6, and TNF-α, which contribute to chronic neuroinflammation in the AD brain [52]. Moreover, p38 MAPK has been implicated in tau hyperphosphorylation and has been found to co-localize with NFTs in the brains of patients with AD [53]. Interestingly, gallic acid, a phenolic acid found in *A. indica*, ameliorated cognitive decline in AD mice by inhibiting the p38 MAPK signaling pathway [54]. These findings suggest that *A. indica* may exert anti-AD effects, at least in part, by suppressing this signaling pathway. KEGG pathway analysis further revealed that the targets of *A. indica* were significantly enriched in neurodegeneration-related pathways, including the Alzheimer’s disease pathway. Several key biomarkers involved in AD pathogenesis have been identified as potential targets of *A. indica* compounds, including genes associated with Aβ pathology, such as *APP* (amyloid precursor protein), *PSEN1/2* (γ-secretase), and *BACE1/2* (β-secretase), as well as tau pathology-related genes, including *MAPT* (tau protein), *GSK3B* (GSK3β), and *CDK5* (cyclin-dependent kinase 5). These targets are well-established in experimental and clinical AD research [55], and their repeated identification in our analysis further strengthens the biological significance of the predicted network pharmacology results. Collectively, these findings suggest that *A. indica* may exert therapeutic effects against AD by modulating multiple molecular targets and signaling pathways.

PPI network analysis identified AKT1, EGFR, IL6, TNF, and TP53 as hub proteins that consistently overlapped across different CytoHubba algorithms, suggesting that these targets may play central roles in the anti-AD potential of *A. indica*. Transcriptomic validation using the GSE33000 dataset further demonstrated that the mRNA expression levels of these genes were significantly elevated in the brain tissues of patients with AD compared to of those healthy controls. In addition, single-gene GSEA indicated that these five hub targets were associated with multiple pathways involved in neurodegeneration and neuroinflammation. Notably, AKT1, EGFR, IL6, TNF, and TP53 are also key components of the PI3K/Akt and MAPK signaling pathways, both of which play crucial roles in AD pathogenesis, as discussed above. This further supports the involvement of these pathways in the underlying mechanisms through which *A. indica* exerts its therapeutic effects against AD. Furthermore, the expression levels of these targets showed strong correlations with alterations in immune cell infiltration within the brain, suggesting that the bioactive compounds of *A. indica* may exert modulatory effects on the neuroimmune system of the brain. Moreover, molecular docking analysis revealed strong interactions between these target proteins and the five potential compounds (chrysin, daidzein, galangin, kaempferol, and quercetin). Among the identified compounds, quercetin exhibited the strongest overall binding affinities toward hub proteins, suggesting that it may play a major role in the therapeutic effects of *A. indica*. Previous studies have demonstrated the therapeutic efficacy and molecular mechanisms of quercetin in AD treatment [56,57]. Quercetin alleviated cognitive impairment by suppressing the production of pro-inflammatory cytokines, including IL-6 and TNF-α, in the hippocampus and prefrontal cortex of scopolamine-induced AD rats [58]. In addition, quercetin exerted neuroprotective effects by inhibiting the MAPK and PI3K/Akt/GSK3β signaling pathways in HT22 neuronal cells [59]. These experimentally validated mechanisms are highly consistent with our docking and network analyses, particularly regarding IL6, TNF, AKT1, and MAPK signaling, further supporting the biological relevance of our computational predictions. Although several clinical studies have demonstrated the anti-inflammatory and antioxidant properties of quercetin, its clinical application in AD has not been thoroughly investigated. Therefore, further clinical studies are needed to evaluate the potential of quercetin and *A. indica* for AD treatment.

This study had several limitations. First, owing to limited computational resources and facilities, the dynamic behavior and stability of the compound–protein complexes were assessed only using CABS-flex 2.0, which is based on coarse-grained protein modeling. Although this approach provides efficient preliminary insights into protein flexibility and conformational dynamics, additional validation using longer and more comprehensive molecular dynamics simulations would further strengthen the reliability of the findings. Another limitation is the lack of experimental validation through in vitro and in vivo studies of the proposed model. Although the integrated bioinformatics analyses provided valuable insights into the potential molecular mechanisms of *A. indica* in AD treatment, further experimental investigations are required to validate these findings.

## 4. Materials and Methods

### 4.1. Compound-Target Network Construction

The chemical composition of *Acalypha indica* was obtained from Dr. Duke’s Phytochemical and Ethnobotanical Database (https://phytochem.nal.usda.gov/, accessed on 15 April 2026), the Indian Medicinal Plants, Phytochemistry, and Therapeutics (IMPPAT) database (https://cb.imsc.res.in/imppat/, accessed on 15 April 2026), and the literature [60,61,62,63]. The collected compounds were combined, and duplicates were removed. General information and SMILES structures of the compounds were obtained from the PubChem database (https://pubchem.ncbi.nlm.nih.gov/, accessed on 15 April 2026) [64]. The Traditional Chinese Medicine Systems Pharmacology Database and Analysis Platform (TCMSP) (https://tcmsp-e.com/tcmsp.php, accessed on 15 April 2026) and SwissTargetPrediction website (https://www.swisstargetprediction.ch/, accessed on 15 April 2026) were used to retrieve the potential targets of the compounds [65,66]. Only targets with a probability value > 0.1 were selected.

GeneCards (https://www.genecards.org/, accessed on 15 April 2026), MalaCards (https://www.malacards.org/, accessed on 15 April 2026), and OMIM (https://www.omim.org/, accessed on 15 April 2026) databases were used to retrieve AD-related targets [67,68,69]. Only targets with a relevance score > 20 in the GeneCards and MalaCards databases were selected for further analysis. Venny 2.1 (https://bioinfogp.cnb.csic.es/tools/venny/, accessed on 15 April 2026) was used to create a Venn diagram showing the overlapping genes between the compounds and AD. A network of drugs and their related AD targets was constructed using Cytoscape version 3.10.4 [70]. Topological analysis was performed to identify the key compounds within the network using the Analyze Network tool in Cytoscape.

### 4.2. Gene Ontology (GO) and Kyoto Encyclopedia of Genes and Genomes (KEGG) Pathway Enrichment Analysis of the Potential Targets

The Database for Annotation, Visualization, and Integrated Discovery (DAVID) 2021 bioinformatics tool (https://davidbioinformatics.nih.gov/, accessed on 17 April 2026) was used to analyze the GO and KEGG pathways associated with potential targets against AD in humans (*Homo sapiens*) [71]. The top 10 GO terms for biological processes, cellular components, and molecular functions, and the top 30 KEGG pathways (*p* < 0.001) were selected and visualized using the SRPlot website (https://www.bioinformatics.com.cn/srplot, accessed on 17 April 2026) [72]. The PI3K-Akt signaling pathway, pathways in neurodegeneration—multiple diseases, and the Alzheimer’s disease signaling pathway were obtained from the KEGG pathway database (https://www.genome.jp/kegg/pathway.html, accessed on 17 April 2026) [73].

### 4.3. Protein–Protein Interaction Network Construction

The STRING database (version 12.0, https://string-db.org/, accessed on 17 April 2026) was used to construct a protein–protein interaction (PPI) network of the potential targets of DE-FRGs in humans (*Homo sapiens*) with a minimum required interaction score of 0.4 [74]. Cytoscape 3.10.4 software with the cytoHubba plugin was used to identify hub targets in the PPI network using five different cytoHubba algorithms (maximal clique centrality (MCC), maximum neighborhood component (MNC), edge percolated component (EPC), Degree, and EcCentricity) and generate a subnetwork of key nodes.

### 4.4. Transcriptomic Validation of Five Hub Genes

The expression levels of the target genes AKT1, EGFR, IL6, TNF, and TP53 were validated using the GSE33000 dataset, which comprised 310 control samples and 157 AD samples [75]. GEO2R was used to analyze the microarray data and compare the normalized gene expression levels between the two groups. To enhance the reliability of the differential expression analysis, *p*-values were adjusted using the Benjamini–Hochberg method to control the false discovery rate (FDR). The diagnostic performance of these biomarkers was further evaluated using receiver operating characteristic (ROC) curve analysis, including the area under the curve (AUC) and corresponding *p*-values.

### 4.5. Single-Gene Gene Set Enrichment Analysis (GSEA)

Single-gene GSEA was conducted using the gseKEGG function in the clusterProfiler package (v4.20.0) with R software (v4.6.0) [76]. To explore the biological pathways related to the five marker genes, correlation analyses were performed between each marker gene and all other genes in the GSE33000 dataset. The ranked gene lists were then analyzed against the KEGG signaling pathway gene set as a reference database for pathway enrichment. Finally, the top five significantly enriched pathways associated with the five hub genes were visualized using the ggplot2 R package (v4.0.3).

### 4.6. Immune Cell Infiltration Analysis

Immune cell infiltration analysis was conducted using the CIBERSORT algorithm in R to estimate the relative proportions of 22 immune cell types in each sample from the GSE33000 dataset [77]. Differences in the proportions of infiltrating immune cell populations between the AD and healthy control groups were assessed using the Wilcoxon test. Pearson’s correlation analysis was performed to explore the associations between the five hub genes and the differentially infiltrated immune cells. The results were visualized using ggplot2 package in R.

### 4.7. Molecular Docking Analysis

AutoDockTools v1.5.7 was used for molecular docking between the potential compounds and top five hub proteins [78]. The three-dimensional (3D) structures of the drugs were downloaded from the PubChem database (https://pubchem.ncbi.nlm.nih.gov/). The protein structures with high crystallographic resolution were retrieved from the Research Collaboratory for Structural Bioinformatics Protein Data Bank (RCSB PDB) (https://www.rcsb.org/, accessed on 25 April 2026) to ensure structural accuracy and reliability for molecular docking analysis [79]. Molecular docking was performed using AutoDock with an energy range of 4 kcal/mol and an exhaustiveness setting of 8. A cubic grid box measuring 40 × 40 × 40 Å (size x, y, z) was used for all the target proteins. The PDB ID and center coordinates (center x, y, and z) of each protein are presented in Table 5. The conformation with the lowest docking score (binding energy) was analyzed using PyMOL v3.1.3 and Discovery Studio Visualizer v25.1.0 for 3D and 2D interaction visualization, respectively [80,81].

### 4.8. Molecular Dynamics Simulation Analysis

Molecular dynamics simulations of the protein–ligand complexes were conducted using CABS-flex V 2.0 (https://biocomp.chem.uw.edu.pl/CABSflex2/, assessed on 10 May 2026) [82]. The simulations were performed for 10 ns using 50 simulation cycles with the global weight parameter set to 1.0. The flexibility of individual amino acid residues in the proteins was analyzed using the root mean square fluctuation (RMSF).

### 4.9. Statistical Analysis

GraphPad Prism v.10 (GraphPad Software, San Diego, CA, USA) was used for statistical analysis. Differences in gene expression levels between the control and AD groups were compared using an unpaired Student’s *t*-test. Statistical significance was set at *p* < 0.05.

## 5. Conclusions

In conclusion, this study represents the first systematic investigation of the potential therapeutic mechanisms of *A. indica* against AD using an integrated bioinformatics approach that combines network pharmacology, pathway enrichment analysis, transcriptomic validation, molecular docking, and dynamic simulation analyses. The findings revealed that *A. indica* contains multiple bioactive compounds, particularly flavonoids and phenolic acids, that may exert anti-AD effects by modulating diverse molecular targets and signaling pathways, especially the PI3K/Akt and MAPK pathways. Among the identified compounds, quercetin exhibited the strongest interactions with the hub proteins (AKT1, EGFR, IL6, TNF, and TP53), highlighting its potential contribution to the therapeutic effects of *A. indica*. Overall, these results provide new insights into the multi-target mechanisms underlying the anti-AD potential of *A. indica* and offer a valuable foundation for future experimental and clinical studies.

## Figures and Tables

**Table 1:** Topological analysis of the top 15 compounds in the compound-target network.

Compound	Degree	Closeness Centrality	Betweenness Centrality	Average Shortest Path Length
Quercetin	128	0.480916	0.316786	2.079365
Kaempferol	80	0.419441	0.084548	2.384127
Daidzein	68	0.406452	0.130733	2.460317
Chrysin	63	0.401274	0.068461	2.492063
Galangin	62	0.400254	0.046716	2.498413
Aurantiamide	52	0.390335	0.140217	2.561905
Asperglaucide	49	0.382746	0.138317	2.612698
Quinine	48	0.382746	0.14436	2.612698
Naringenin	42	0.379976	0.05219	2.631746
Ellagic acid	41	0.379061	0.047314	2.638095
Hesperetin	40	0.379061	0.036221	2.638095
Rosmarinic acid	38	0.376344	0.059585	2.657143
Epicatechin	36	0.374554	0.034558	2.669841
Stigmasterol	30	0.365006	0.035056	2.739683
Catechin	23	0.363322	0.007947	2.752381

**Table 2:** Molecular docking interaction profiles of representative protein–compound complexes.

Complex	Binding Energy (kcal/mol)	Interaction Type	Residue	Distance (Å)
Galangin–AKT1	−8.2	Pi-Anion	ASP292	3.38
		Pi-Anion	GLU198	3.98
		Pi-Cation	LYS179	2.94
		Pi-Pi T-shaped	PHE161	5.01
		Van der Waals	GLY159, THR160, LEU181, GLU191, HIS194, THR195, ASP274, LYS276, GLY294, LEU295, THR312	–
Chrysin–EGFR	−7.6	Conventional hydrogen bond	ARG817	2.11
		Pi-Pi stacked	PHE699	4.33
		Pi-Pi stacked	PHE699	4.56
		Pi-Anion	ASP831	3.49
		Pi-Anion	ASP831	3.96
		Van der Waals	VAL702, LYS721, CYS773, ASN818, LEU820	–
Quercetin–IL6	−6.5	Conventional hydrogen bond	GLU95	2.86
		Conventional hydrogen bond	LYS120	2.58
		Conventional hydrogen bond	GLN116	2.05
		Conventional hydrogen bond	PRO139	2.30
		Conventional hydrogen bond	ASN144	2.47
		Carbon hydrogen bond	THR138	3.33
		Pi-Anion	GLU95	3.85
		Pi-Anion	GLU95	3.97
		Pi-Anion	GLU99	3.53
		Pi-Cation	LYS120	2.91
		Unfavorable donor-donor	ASN144	1.42
		Van der Waals	LEU92, VAL96, LEU98, PRO141	–
Quercetin–TNF	−8.6	Conventional hydrogen bond	LEU93 (chain D)	2.76
		Conventional hydrogen bond	GLN125 (chain B)	2.35
		Conventional hydrogen bond	GLN125 (chain D)	1.79
		Pi-Alkyl	VAL91 (chain B)	5.09
		Pi-Alkyl	VAL91 (chain D)	5.25
		Pi-Alkyl	LEU93 (chain B)	5.44
		Van der Waals	ARG82 (chain B, D), ASN92 (chain B, D), LEU94 (chain D), PHE124 (chain B, D), LEU126 (chain D)	–
Quercetin–TP53	−8.9	Conventional hydrogen bond	ASN247 (chain B)	1.91
		Conventional hydrogen bond	LYS139 (chain C)	2.25
		Conventional hydrogen bond	LYS139 (chain C)	2.47
		Conventional hydrogen bond	THR123 (chain C)	1.98
		Conventional hydrogen bond	GLU198 (chain C)	2.45
		Pi-Alkyl	LYS139 (chain C)	4.76
		Pi-Anion	GLU171 (chain B)	4.32
		Pi-Cation	ARG249 (chain B)	4.23
		Pi-Cation	ARG249 (chain B)	4.33
		Pi-Cation	HIS168 (chain B)	3.66
		Pi-Cation	HIS168 (chain B)	4.45
		Pi-Pi T-shaped	HIS168 (chain B)	4.61
		Unfavorable donor-donor	THR140 (chain C)	2.05
		Van der Waals	GLN167 (chain B), THR170 (chain B)	–

**Table 3:** Maximum and minimum RMSF values and corresponding residues of target proteins.

RMSF Parameter		AKT1	EGFR	IL6	TNF	TP53
Maximum	Value (Å)	4.142	13.102	3.953	4.752	4.664
	Residue	A439	A995	A61	D21	C95
Minimum	Value (Å)	0.081	0.106	0.088	0.045	0.046
	Residue	A177	A876	A91	A155	B271

**Table 4:** ADMET prediction of potential compounds in *A. indica* for AD.

Parameter		Chrysin	Daidzein	Galangin	Kaempferol	Quercetin
Absorption	Caco2 permeability (log P_app_)	0.945	0.903	0.999	0.032	−0.229
	Intestinal absorption (%)	93.761	94.839	93.985	74.29	77.207
Distribution	VDss (human) (log L/kg)	0.403	−0.172	0.816	1.274	1.559
	Fraction unbound (human)	0.136	0.107	0.142	0.178	0.206
	BBB permeability (log BB)	0.047	−0.064	−0.748	−0.939	−1.098
	CNS permeability (log PS)	−1.912	−1.992	−2.068	−2.228	−3.065
Metabolism	CYP1A2 inhibitor	Yes	Yes	Yes	Yes	Yes
	CYP2C19 inhibitor	Yes	Yes	Yes	No	No
	CYP2C9 inhibitor	Yes	Yes	Yes	No	No
	CYP2D6 inhibitor	No	No	No	No	No
	CYP3A4 inhibitor	No	No	No	No	No
Excretion	Total clearance (log mL/min/kg)	0.405	0.164	0.256	0.477	0.407
	Renal OCT2 substrate	No	No	No	No	No
Toxicity	Ames toxicity (mutagenicity)	No	No	No	No	No
	Hepatotoxicity	No	No	No	No	No
	Max. tolerated dose (human) (log mg/kg/day)	0.016	0.187	0.333	0.531	0.499

P_app_, apparent permeability coefficient; VDss, volume of distribution at steady state; BBB, blood–brain barrier; BB, brain-to-blood concentration ratio; CNS, central nervous system; PS, permeability–surface area product; CYP, cytochrome P450; OCT2, organic cation transporter 2.

**Table 5:** The center coordinates (center x, y, and z) of the hub proteins.

Protein	PDB ID	Resolution (Å)	Center x (Å)	Center y (Å)	Center z (Å)
AKT1	4EKL	2.00	−4.302	1.855	−11.226
EGFR	1M17	2.60	23.553	9.725	59.329
IL6	1ALU	1.90	2.523	−19.96	8.684
TNF	2AZ5	2.10	−13.680	71.594	26.989
TP53	1TUP	2.20	59.456	12.218	77.813

## Data Availability

The raw data supporting the conclusions of this article will be made available by the authors on request.

## Supplementary Materials

The following supporting information can be downloaded at https://www.mdpi.com/article/10.3390/ijms27146196/s1.

## Abbreviations

The following abbreviations are used in this manuscript:

AD	Alzheimer’s disease
Aβ	Amyloid beta
NFT	Neurofibrillary tangles
IMPPAT	Indian Medicinal Plants, Phytochemistry, and Therapeutics
TCMSP	Traditional Chinese Medicine Systems Pharmacology Database and Analysis Platform
KEGG	Kyoto Encyclopedia of Genes and Genomes
GO	Gene Ontology
BP	Biological process
CC	Cellular component
MF	Molecular function
PI3K/Akt	Phosphatidylinositol 3-kinase/protein kinase B
MAPK	Mitogen-activated protein kinase
PPI	Protein–protein interaction
MCC	Maximal clique centrality
MNC	Maximum neighborhood component
EPC	Edge percolated component
EGFR	Epidermal growth factor receptor
IL6	Interleukin 6
TNF	Tumor necrosis factor
TP53	Tumor protein p53
ROC	Receiver operating characteristic
GSEA	Gene set enrichment analysis
NK	Natural killer
RMSF	Root mean square fluctuation
ADMET	Absorption, distribution, metabolism, excretion, and toxicity
