# Supplementary material for: Investigation of the Potential Neuroprotective Mechanisms of Acalypha indica Against Alzheimer’s Disease by Integrated Bioinformatics Analysis
Source: Int J Mol Sci. 2026 Jul 11;27(14):6196. doi: 10.3390/ijms27146196 (PMC13409892; doi:10.3390/ijms27146196)
Supplement: Supplementary file 1 [file ijms-27-06196-s001.zip › Supplementary Figures.pdf]

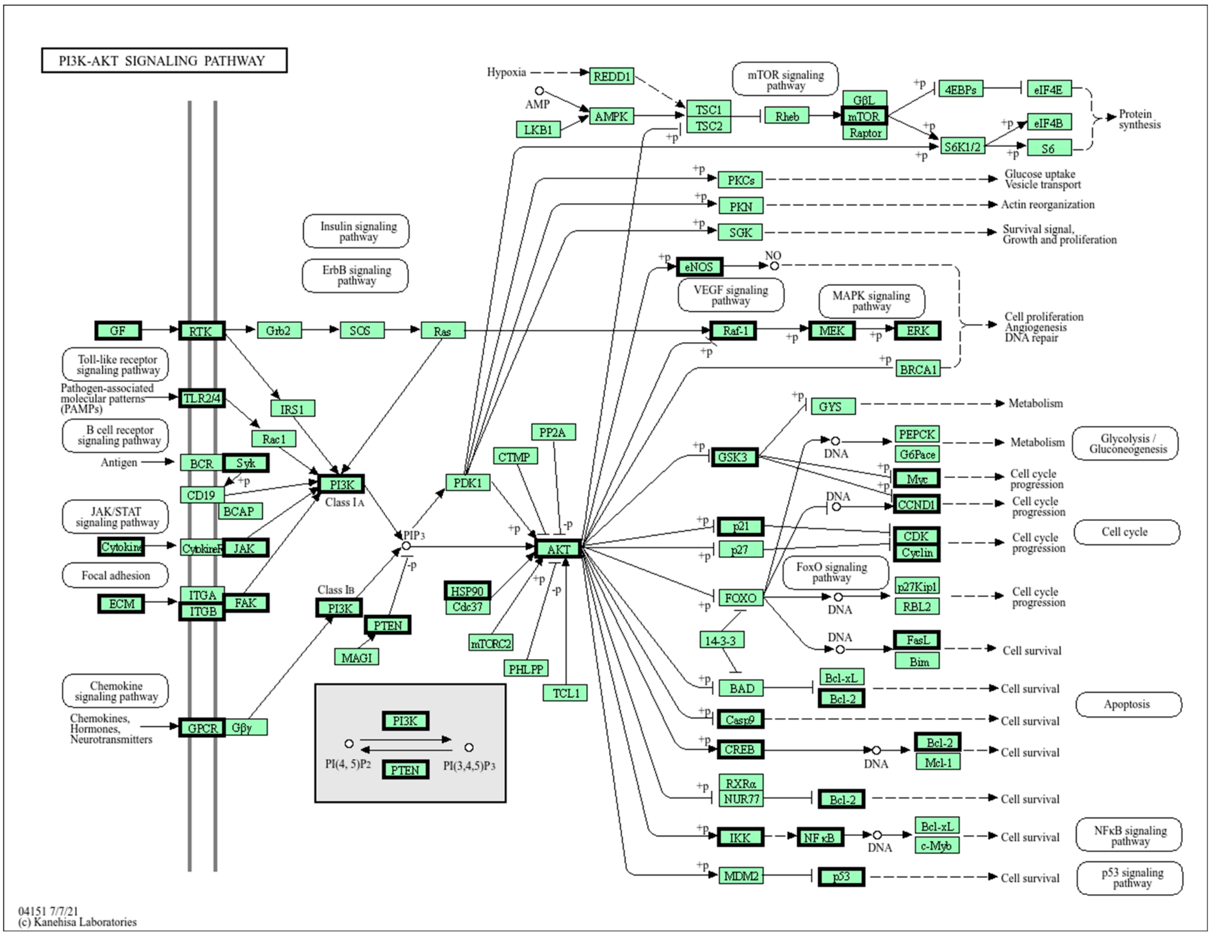

Figure S1. PI3K-Akt signaling pathway. Potential targets of *A. indica* are highlighted with bold borders.

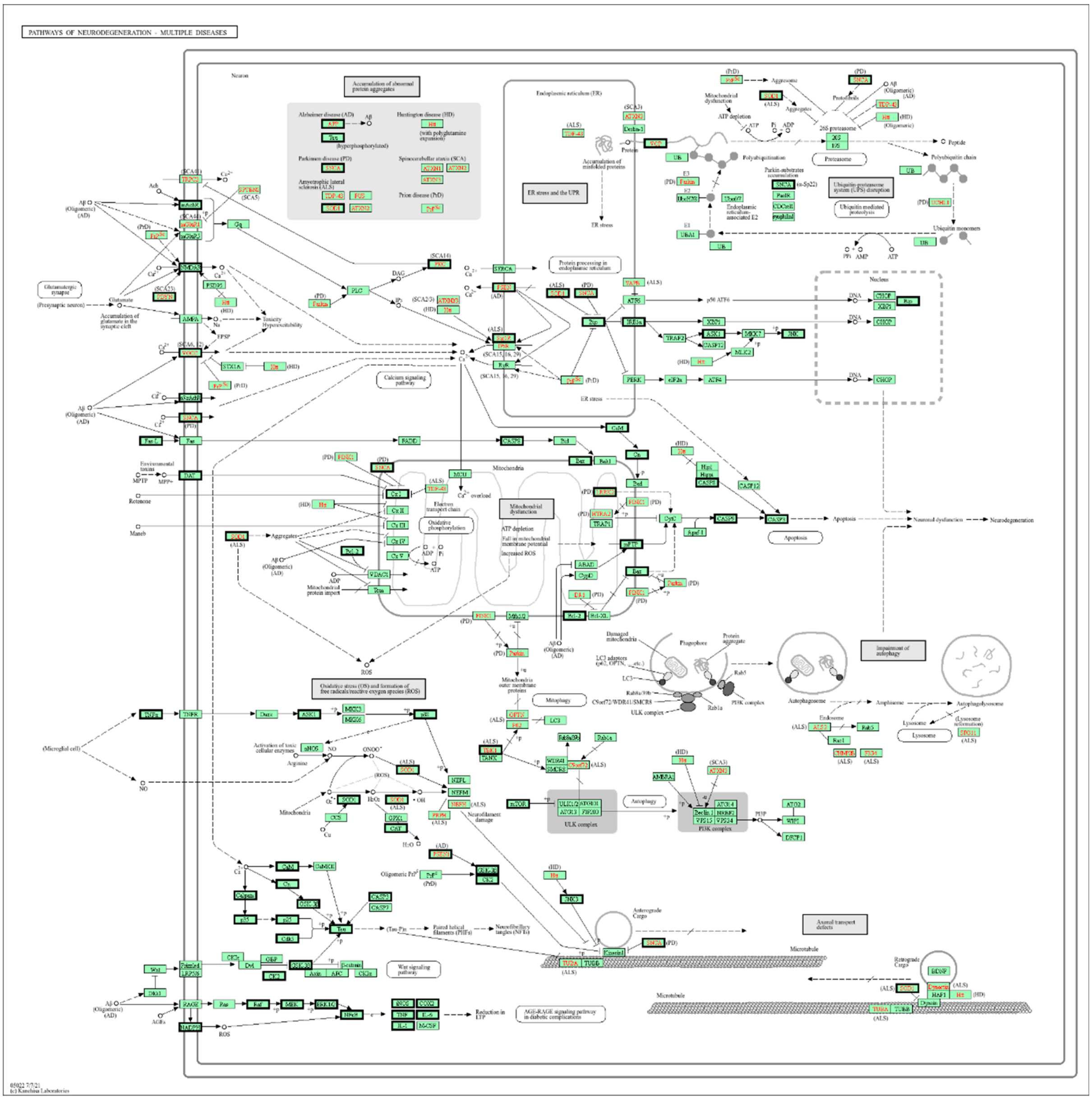

Figure S2. Pathways of neurodegeneration - multiple diseases. Potential targets of *A. indica* are highlighted with bold borders.

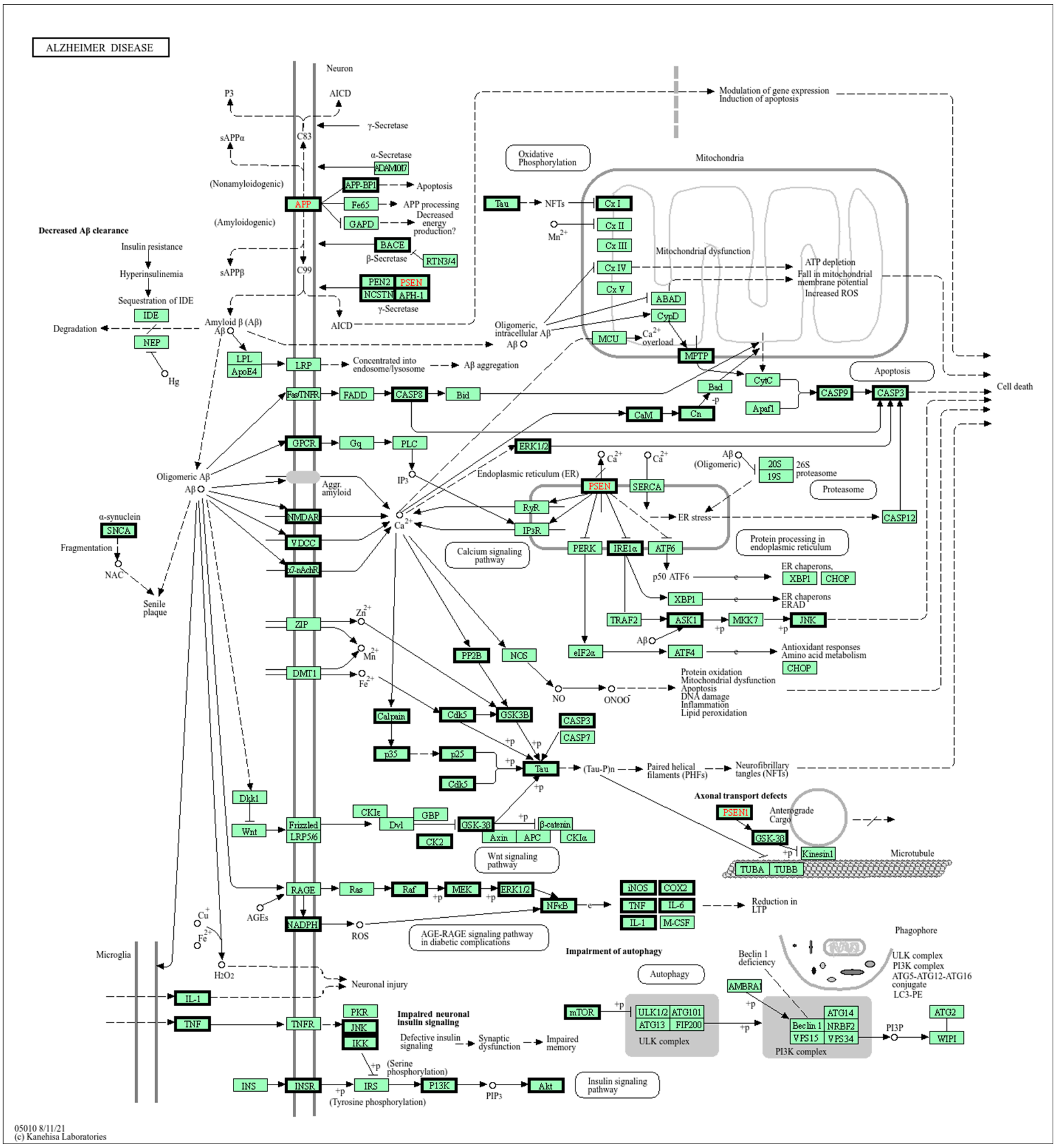

Figure S3. Alzheimer disease. Potential targets of *A. indica* are highlighted with bold borders.

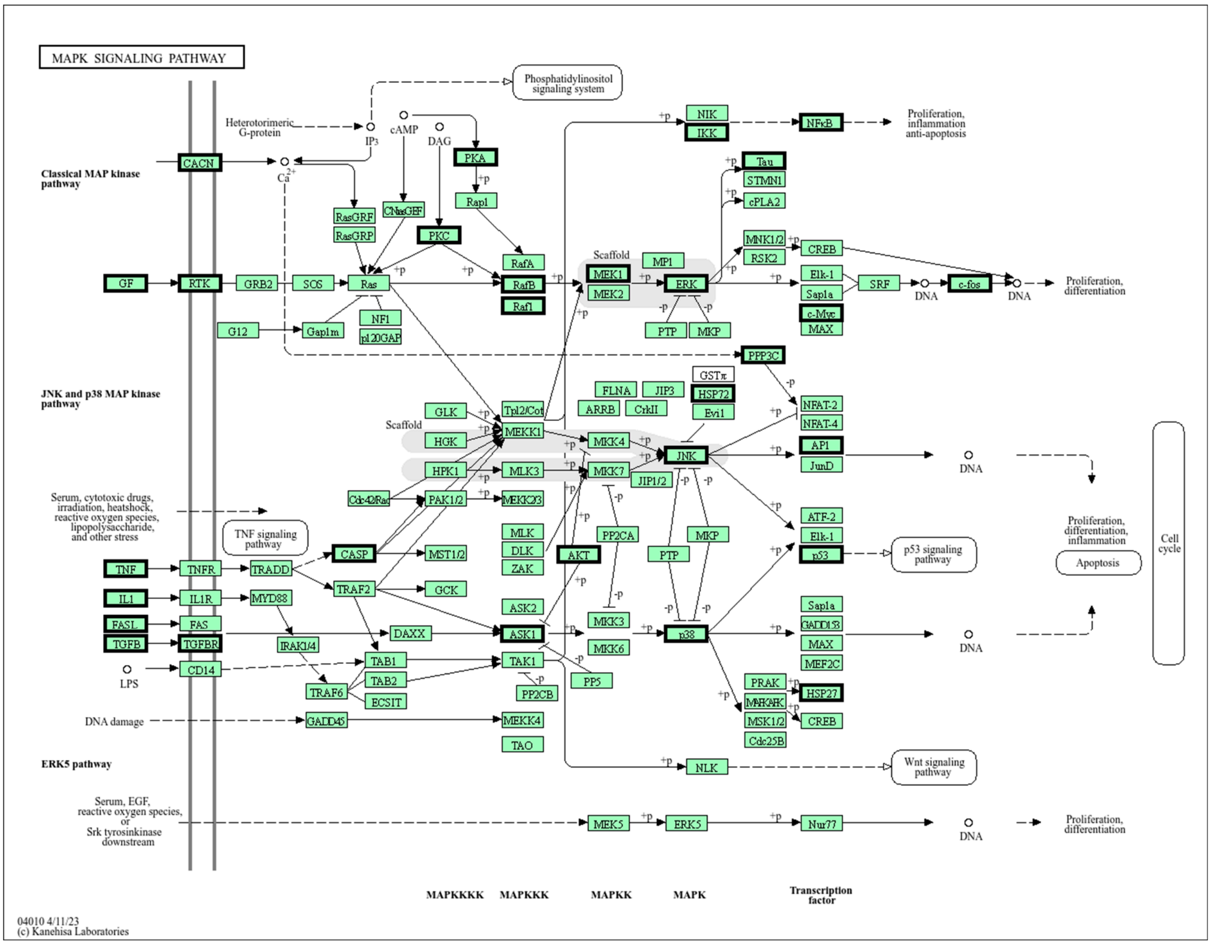

Figure S4. MAPK signaling pathway. Potential targets of *A. indica* are highlighted with bold borders.
